# From Materials to Technique: A Complete Non-Invasive Investigation of a Group of Six Ukiyo-E Japanese Woodblock Prints of the Oriental Art Museum E. Chiossone (Genoa, Italy)

**DOI:** 10.3390/s22228772

**Published:** 2022-11-13

**Authors:** Marco Gargano, Margherita Longoni, Valeria Pesce, Maria Chiara Palandri, Aurora Canepari, Nicola Ludwig, Silvia Bruni

**Affiliations:** 1 Dipartimento di Fisica, Università degli Studi di Milano, Via G. Celoria, 16, 20133 Milan, Italy; 2 Dipartimento di Chimica, Università degli Studi di Milano, Via C. Golgi, 19, 20133 Milan, Italy; 3 Accademia di Belle Arti di Brera, Via Brera 28, 20121 Milan, Italy; 4 Department Collections and Research, National Library of Norway, Henrik Ibsens Gate 110, 0255 Oslo, Norway; 5 Museo d’Arte Orientale E. Chiossone, Piazzale G. Mazzini 4, 16122 Genova, Italy

**Keywords:** Japanese woodblock print, multiband imaging, UV fluorescence, visible induced luminescence, reflectance transformation imaging, Raman spectroscopy, reflectance FTIR spectroscopy, spectrofluorimetry, fiber optic reflectance spectroscopy

## Abstract

In the present work, a complete non-invasive scientific investigation of six Utagawa Kunisada’s woodblock prints (*nishiki-e*) belonging to the Oriental Art Museum “E. Chiossone” (Genoa, Italy), was performed in situ. The campaign started with high resolution multiband imaging (visible, multiband fluorescence, near infrared) followed by reflectance transformation imaging (RTI) to characterize and highlight the peculiar printing techniques and the condition of the support. Then fiber optics reflectance spectroscopy (FORS), spectrofluorimetry, Raman and reflectance Fourier-transform infrared (FTIR) spectroscopies were successfully applied in synergy for the investigation of the printing materials (pigments, binders, support). The results obtained represent a set of very important information for these never-before-studied works of art, useful to the different professionals involved: historians, conservators and curators. The materials identified were completely in agreement with those traditionally used in the Edo period in the 19th century, while the computational imaging technique RTI gave an additional amount of information in terms of surface characterization that could not be overlooked when studying these works of art. RTI data were further processed to enhance the texture visualization.

## 1. Introduction

*Ukiyo-e* printing is a traditional Japanese multicolor woodblock printing style that flourished during the 18th century. The Japanese printing technique *mokuhanga*, literally “print (*hanga* 版画) on wood (*moku* 木)”, is undeniably the artistic expression which has led to its diffusion and affirmation all over the world. Similar to the woodcut process known in Europe, this method allows the multiple reproduction of images on paper. However, the Japanese system followed its own evolution, so that the *mokuhanga* distinguished itself as an artistic technique, unique of its kind.

Since the mid 60s of the eighteenth century, the Japanese technique of printing from woodblocks developed thanks to experimentation of its best-known form: the “brocade prints” (*nishiki-e*). Such a phenomenon occurred in response to growing public demand as well as the development of Japanese publishing [1]. It should be remembered, in fact, that *ukiyo-e* woodblock prints have a very distinct commercial nature, and their production expanded during the Edo period (1603–1868) thanks to the synergy of different figures: the publisher, the artist, as well as professional block carvers and printers.

The inks used for traditional Japanese woodblock printing were water-based colors. Ready to use, the color was brushed onto the damp block surface and combined with rice or wheat starch paste. The ink absorption through the paper fibers was controlled by sizing the paper surface with *dōsa*, a mixture of animal glue (*nikawa*) and alum. The *nikawa* was also commonly employed as a binder for pigments. The protein-based adhesive could be added to the powder colors, forming solid inks to be dispersed in water for use. The color palette of Japanese woodblock prints could include different pigments as well as natural or synthetic dyes. Many studies have suggested specific time windows regarding their use, or non-use, in the Edo (1603–1868) and Meiji (1868–1912) periods, making it possible to reconstruct a timeline of the evolution of the coloring materials used in *ukiyo-e* prints [2,3,4].

To achieve the desired tone, these traditional inks were sometimes mixed and/or overprinted on each other. Their combination could also imply the use of different materials with the same hue [1]. Each block (color) could be printed in more than one impression, in order to reach a more and more saturated result. The image obtained, at the end of the entire printing process, was the consequence of multiple impressions of a set of blocks to one single sheet of paper. *Ukiyo-e* prints were indeed enriched by special effects such as gradations (*bokash*i techniques) or embossing (*karazuri*, *kimedashi* and *nunomezuri* techniques). The *karazuri* and the *kimedashi* employed separate woodblocks, cut in the shape required for the blind-printed impression. They both gave to ukiyo-e prints three-dimensional details, but in the case of *kimedashi,* the paper was pushed down into the carved spaces of the block with a tool different from the traditional circular pad, known as a *baren*, so that the embossed texture appeared more convex and less deep. The *Nunomezuri* technique required, instead, a piece of fabric glued to a woodblock carved shape. The pressure applied by the *baren* ensured that the texture of the fabric was embossed throughout that specific shape [5].

In this scenario, the identification of the materials used to realize Japanese woodblock prints proved to be indispensable not only for the deep understanding of these objects and of this complex technique, but also for the assessment of specific critical issues related to their conservation [6,7].

Several scientific methods have been proposed in the literature for studying the materials of Japanese woodblock prints. Since the risk of damaging the appearance of this type of objects can be very high, they are considered too fragile to allow sampling and non-invasive techniques are preferred for their investigation. As far as we know, only a few studies involving destructive techniques have been reported, namely liquid chromatography with mass spectrometry (HPLC-MS) and surface-enhanced Raman spectroscopy (SERS) [4,8]. As a consequence, imaging and spectroscopic techniques are the most adopted. Multispectral imaging has been used several times to study Japanese woodblock prints, providing useful information on the distribution of painting materials. Furthermore, if combined with single-point spectroscopic analytical methods, this technique can give reliable results in terms of identifying inorganic pigments and, to some extent, organic dyes. The most common coupling is with fiber-optic reflectance spectroscopy (FORS) and/or X-ray fluorescence (XRF) [3,4,9,10,11,12,13], although also the combined use of spectrofluorimetry has been reported [14,15]. Moreover, again referring to single-point spectroscopic methods, the application of Fourier-transform infrared (FTIR) [3,15,16] and Raman spectroscopy [4,8,17] was also documented for the study of woodblock prints. It is worth noting that these latter techniques have the great advantage of providing more specific information on the analytes as they are vibrational spectroscopies. However, their application can be challenging: FTIR spectra can be dominated by the signal due to the paper substrate and/or the binder, while Raman spectroscopy is the ideal tool for the identification of inorganic pigments, but it is affected by the problem of fluorescence in the case of organic materials, such as dyes.

In the present work, a complete non-invasive scientific investigation was performed as part of the conservation project of six woodblock prints designed by the Japanese artist Utagawa Kunisada (1786–1865), recently donated to the Oriental Art Museum E. Chiossone by Prof. Madeleine Cavalier, widow of the Genoese archeologist Luigi Bernabò Brea (1910–1999) [18]. These artworks were part of a larger collection donated to the Museum with the aim of preserving it and making it accessible to the public, promoting the planning of a specific conservation project.

The project was aimed at guiding the conservation intervention and characterizing the materials and the techniques of the *ukiyo-e* woodblock prints, as well as their conservation conditions. The investigations were carried out in situ in the Museum venue and started with high resolution multiband imaging (visible, multiband fluorescence, near infrared) followed by reflectance transformation imaging (RTI) to characterize and highlight the peculiar printing techniques and the condition of the support. Then, as single-point techniques for non-invasive analysis, FORS, reflectance FTIR spectroscopy, Raman spectroscopy and spectrofluorimetry with visible excitation were used to achieve a complete characterization of the coloring materials used by the artist. In particular, for the fluorescence measurements, the use of visible excitation radiation with wavelengths suitable for the different hues was chosen in order to obtain a higher selectivity towards the coloring matter itself. We emphasize that an innovative multiband visible fluorescence imaging technique was applied to compare and extend the spot fluorescence analysis, particularly useful when dealing with organic dyes and pigments [19]. As regards the Raman spectroscopic measurements, a spectrometer based on sequentially shifted excitation (SSE) technology was used, to limit as far as possible the contribution of the fluorescence background due to the support, the binder and the colorants themselves.

## 2. Materials and Methods

### 2.1. The Woodblock Prints by Utagawa Kunisada

The woodblock prints studied in the present work belong to the collection of Japanese *ukiyo-e* prints of the Edoardo Chiossone Museum of Oriental Art in Genoa (Italy). The collection, one of the largest and finest in Europe, was donated to the community by the Italian engraver Edoardo Chiossone, who lived and worked in Japan for 23 years during the Meiji period.

The six prints under investigation were made by Utagawa Kunisada, one of the most popular and productive designers of 19th century Japanese woodblock prints. Kunisada, also known as Toyokuni III, became a pupil of Toyokuni I around 1800. During his long and prolific career, he distinguished himself as one of the main figures of the Utagawa school. Kabuki actors and beautiful women were among his most famous and notable subjects [20]. The prints examined in the present work are listed in Table 1.

### 2.2. Multiband Imaging

**Figure 1:**
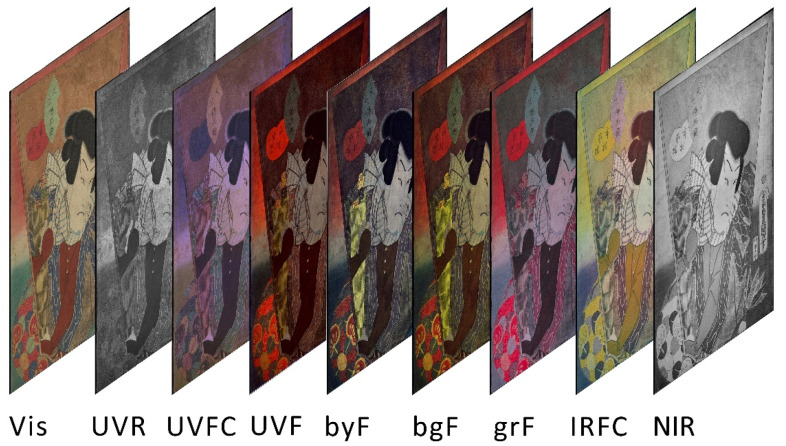
Schematic representation of all the multiband imaging acquisitions performed on each woodblock print as detailed in Table 2.

Visible and multiband luminescence images were acquired with a photographic system consisting of digital back Phase One IQ3, with “Trichromatic” (100 MP) detector (16-bit RAW files of 11,608 × 8708 pixels), able to detect radiation from 350 to 1000 nm. The scheme of the multiband acquisition is shown in Figure 1. For all the acquisitions, a Schneider Kreuznach 120 mm LS f/4.0 Macro lens was used in combination with different filters and light sources to select the appropriate wavelength range for the investigation. The camera was placed horizontally on a solid portable repro tripod. To obtain a high-resolution image of the artworks, two images were shot for each woodblock print, obtaining a final image of 15,300 × 11,500 pixels with a spatial resolution of 46 pixel/mm.

The lighting was chosen according to the different purposes, and different filters were mounted on the lens to correctly acquire the images as described below:

- Visible images (Vis): two Godox Witstro AD360 flashlights (360 W with light diffuser) were used for the visible images, placed symmetrically at 45° to the painting surface. UV/IR cut filters were mounted on the lens to select radiation in the visible range.
- UV reflected images (UVR): two 365 nm (±10 nm, 3 W power) UV LED lamps (Madatec Srl, Milan, Italy) were used as UV light sources applying a 300–400 nm bandpass filter.
- UV false color images (UVFC): Vis and UVR images were combined using the blue and green channels of the Vis image, shifted, respectively, to the green and red channels of the UVFC image, and the UVR image was used in the blue channel.
- Visible fluorescence induced by UV radiation (UVF): the woodblock prints were irradiated with two 365 nm (±10 nm, 3 W power) UV LED lamps (Madatec Srl, Milan, Italy). A combination of a long-pass filter (cut-on wavelength of 420 nm) and a UV/IR cut filter was used to reduce the possible reflection by the artwork of the blue component emitted by the UV sources.
- Visible fluorescence induced by visible radiation (VIVF): different LED light sources with emitted wavelengths of 400, 440, and 530 nm (±10 nm, 3 W power) (Madatec Srl, Milan, Italy) were combined, respectively, with 480, 520 and 600 nm long-pass filters, used to exclude the reflected part of the excitation radiation.
- Near Infrared reflected images (NIR): two Godox Witstro AD360 flashlights (360 W with light diffuser) placed symmetrically at 45° to the painting surface were used with a 850 nm long-pass filter mounted on the lens of the camera.
- IR False Color images (IRFC): Vis and NIR image were combined using the green and red channels of the Vis image, shifted, respectively, to the blue and green channels of the IRFC image, and the NIR image was used in the red channel.

Table 2 summarizes the acquisition methods with specified excitation (λ_exc_) and acquisition (λ_acq_) wavelengths.

### 2.3. Reflectance Transformation Imaging

RTI is an imaging technique used for estimating the intensity and direction of the light reflected from an object, with the aim of representing that object under different directions of the incident light, through the interactive re-lighting of the subject [21,22,23]. RTI images are created with multiple photographs of the subject shot from a fixed camera position and different but known light directions. The object is then represented by a series of images with varying highlights and shadows that are used to generate a geometrical model of the surface using the normal map rendering. For each woodblock print, 100 images were acquired corresponding to 100 different light positions obtained by moving manually the flashlight source (Godox Witstro AD360) and using wireless remote controllers. With this set-up, it was possible to cover uniformly the hemisphere around the object. Each image set was processed using RTI builder software (Version 2.0.2) and the file was visualized interactively with RTI viewer [24]. This last software is also able to give the normal map image [25] that contains all the 3D information using a 2D RGB image and that can be subsequently processed to enhance all the print features.

### 2.4. Reflectance FTIR Spectroscopy

For FTIR non-invasive analysis in reflection mode, a Bruker Alpha FTIR spectrophotometer was used. The instrument is provided with a reflection module for contactless measurements and a deuterated triglycine sulfate (DTGS) detector, operating at room temperature and ensuring a linear response in the spectral range between 7500 and 375 cm^−^^1^. Spectra were collected as a sum of 200 scans, after the acquisition of a background spectrum on a gold mirror. The area of the sample investigated had a diameter of approximately 6 mm and the instrumental resolution was 4 cm^−^^1^. An integrated camera allows the operator to select the area to be measured. The reflection spectra in the MIR region, if necessary, were processed by the Kramers–Kronig transform (KKT) using the Bruker OPUS software (Version 6.5). Alternatively, they were transformed into pseudo-absorbance Log(1/R).

### 2.5. Raman Spectroscopy

Raman analyses were performed in situ using a Bruker BRAVO handheld spectrometer, based on the patented SSE technology, which allows the mitigation of the fluorescence background. This instrument exploits, for the excitation of spectra, two diode lasers operating at different temperatures and emitting, respectively, at 850 and 785 nm. An appropriate algorithm allows the extraction of the final Raman spectral data. The spectra are collected in two sequential steps, from 300 to 2000 cm^−1^ and from 2000 to 3200 cm^−^^1^. The average spectral resolution is approximately 11 cm^−1^. The applied laser power was less than 100 mW for both lasers and the beam was focused on a small rectangular area of approximately 500 × 100 μm, so the power density was really limited and the risk of damage to the investigated artwork reduced. The acquisition time and the number of accumulations were automatically set by the instrument and the first ranged from 0.4 to 3 s while the second from 1 to 15.

### 2.6. Spectrofluorimetry

Spectrofluorimetric measurements were performed by means of a portable microprobe equipped with an Olympus 20× objective and connected by optical fibers to a halogen source (maximum power 150 W) and to a Lot Oriel MS125 spectrometer (grid 400 lines/mm) provided with an Andor CCD detector (1024 × 128 pixel) cooled by means of a Peltier device. The wavelength calibration was based on the emission spectrum of a neon lamp. An interference filter centered at 435 nm (for yellow and red dyes) or 562 nm (for blue dyes) was used to select the wavelength of the incident radiation and a dichroic filter with transmission range 458–700 nm or 635–890 nm, respectively, allowed us to eliminate from the spectrum the component due to the exciting radiation. The measurement spot had a diameter of 4 mm. The spectra were collected as a sum of 30 scans with an exposure time of 2 s.

### 2.7. Fiber Optic Reflectance Spectroscopy (FORS)

A portable Vis–NIR spectrophotometer (HR4000, Ocean Optics, Dunedin, FL, USA) was used for FORS analysis, with a linear array detector, 360–1100 nm measurement range, 2.7 nm spectral resolution, a 45°/45° geometry of measurement and a detection area with a diameter of about 2 mm. The spectrophotometer was connected to a tungsten halogen light source (D65, HL2000, Ocean Optics): light was transmitted through a quartz fiber optics bundle 1.5 m long (Ocean Optics), composed of six fibers (400 μm each), to collect reflected light around the single central illuminating fiber (400 μm). The spectrometer was connected to a laptop and calibrated using white and black reflectance standards (Spectralon 99% and dark trap). The visible–NIR reflectance spectrum from 380 to 1000 nm was recorded for each sample.

## 3. Results and Discussion

### 3.1. High Resolution Multiband Imaging

**Figure 2:**
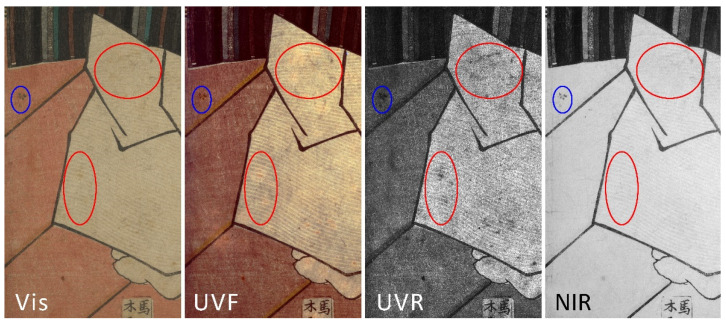
Detail of print 3a showing that, through a multiband acquisition using Vis, UVF, UVR and NIR, it is possible to provide a complete mapping of decay, such as stains (blue circles) and discolorations (red circles).

The general results obtained through high resolution imaging techniques allowed us firstly to evaluate the conservation conditions of the woodblock prints. In this part of the study, the most useful imaging techniques were UVF and NIR, able to highlight different conservative issues. Figure 2 reports the detail of print 3a where the combination of UVF, UVR and NIR acquisitions allowed us to visualize various types of stains, discoloration and decay, not noticeable with a single technique. Blue and red circles in Figure 2 represent different types of decay: blue circles highlight carbon-based stains referable to the printing process, that are visible in all the bands considered. Red circles, instead, represent a decay not visible in the NIR but well highlighted in the UVF and even more in the UVR. This result demonstrates the need for a multiband approach when dealing in general with paper-based material, but even more with this specific type of objects.

**Figure 3:**
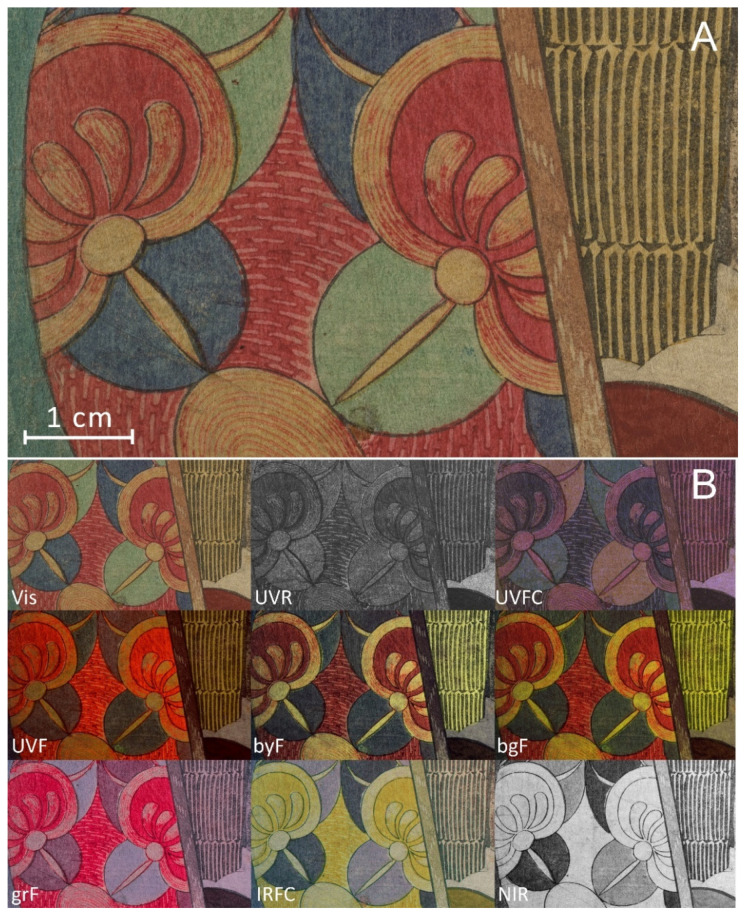
Detail of print 1 showing the high resolution of the acquisition (**A**) and a general view of all the multiband images (**B**).

Together with the ability to visualize the details in different bands, there was also the requirement of high resolution in detail documentation. As specified in Section 2.2, all the images had a spatial resolution of 46 pixel/mm, allowing precise examination of the printing techniques, also, in terms of possible misalignments in the registration of the paper on the different woodblocks. In some cases, it was not only possible to visualize with high detail the graphical details of the printing as a result of using the different matrices (Figure 3A) but also the succession of color layers due to the relative overlap with the other layers, as in the images shown in Figure 3B.

### 3.2. Reflectance Transformation Imaging

**Figure 4:**
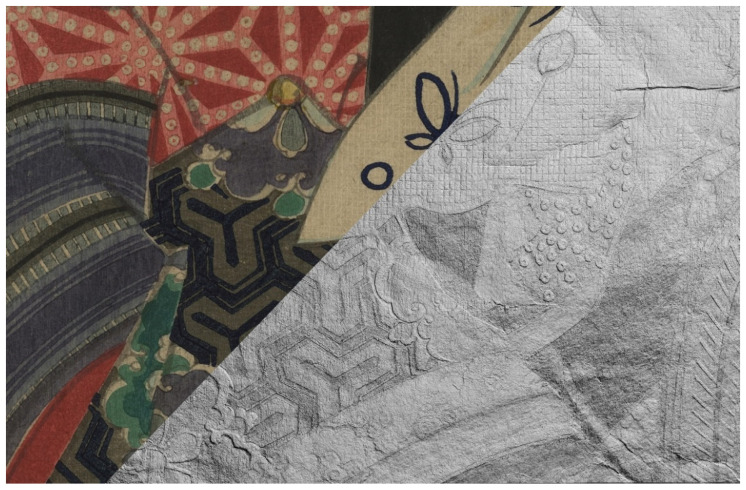
RTI detail of print 4 showing the embossed features, as a result of the different printing techniques, compared to the visible image.

The RTI technique is particularly suitable for documenting and characterizing the three-dimensional features of the woodblock prints as a result of the pressure applied by the *baren* to transfer the ink from the woodblock to the paper. Together with the interactive re-lighting of the object under different light directions, it was possible to emphasize particular features linked to both printing technique and the conservation conditions. Figure 4 represents a detail of print 4 split in two: in the upper left corner, there is the Vis image; in the lower right corner, a processed normal map image to emphasize the 3D features. The apparently flat surface of this detail revealed its very peculiar three-dimensional nature, where all the colored areas had strong embossed features. Furthermore, it was possible to highlight and distinguish specific printing techniques among the many possible that were used to enhance the design [5]: for example, the *karazuri* printing technique, literally “empty printing” (Figure S1) and the *nunomezuri* printing technique (Figure S2).

**Figure 5:**
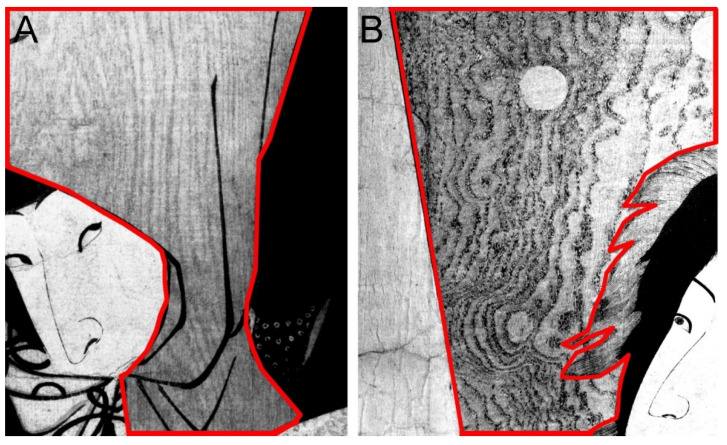
NIR detail of print 4 (**A**) and print 2 (**B**) showing in red outline the enhanced pattern of different woodblocks, typical of the *Mokumezuri* printing technique.

In addition to these two techniques, it is worth including the *mokumezuri* printing technique, literally “wood-eye printing”, that consists in making visible the wood grain patterns using different woods for the blocks. In Figure 5, two details, respectively, of print 4 and print 2, obtained through the NIR technique, allowed us to enhance the wood pattern of two different wood species: wild cherry tree woodblock in print 4 and paulownia tree in print 2.

### 3.3. Spectroscopic Analyses

The results of the single-point spectroscopic analyses, FORS, Raman, reflectance FTIR and spectrofluorimetry, are reported below.

From a general point of view, it should be noted that information about the coloring matter could be gathered mainly by FORS, Raman and spectrofluorimetric measurements. Indeed, reflectance FTIR spectra were mostly dominated by signals associated with a polysaccharide material, at 1626, 1424, 1366, 1315, 1166, 1114, 1061 and 894 cm^−1^ (Figure S3), having the best match with the FTIR spectrum of cellulose [26]. Of course, the contribution to the FTIR spectra of chemically similar materials, such as the rice flour paste usually mixed with the colorant before applying it to the woodblock [27], as already reported above, cannot be excluded. However, the overall spectral pattern of rice starch, dominated by a band located at 1021 cm^−1^, as well as the positions of its bands [28,29] are different from those observed in the present investigation. Consequently, it can reasonably be assumed that the FTIR bands detected for the prints studied were mainly due to their paper support, possibly overlapping those due to a polysaccharide binder. Interestingly, some of the spectra, in particular those associated with non or lightly colored parts (such as a character’s face), also show a rather broad band around 980–975 cm^−1^, possibly due to clay used as a filler to improve the opacity and smoothness of the paper [1].

The inorganic and organic colorants identified by FORS, Raman and spectrofluorimetric analyses in the examined prints are summarized in Table 3 and discussed below according to the different colors.

#### 3.3.1. Red Colors

**Figure 6:**
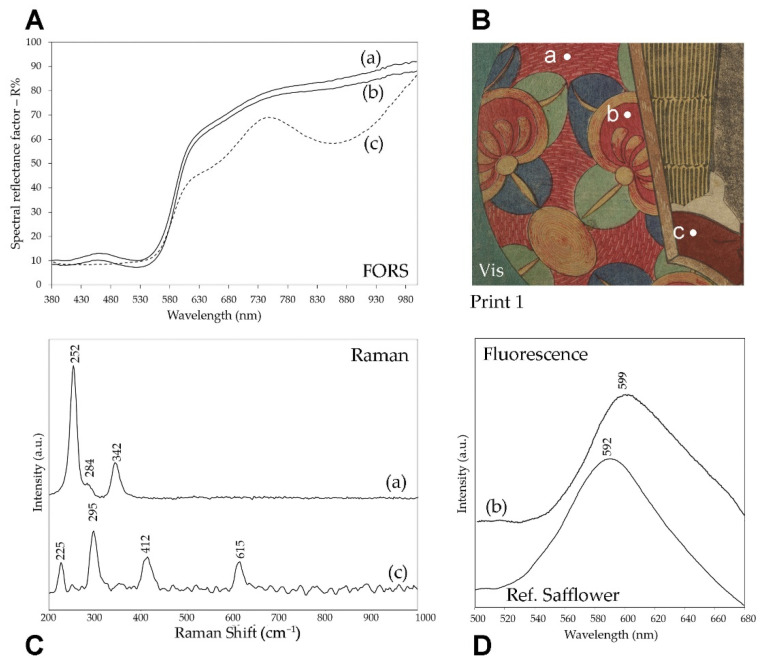
Red details in print 1: (**A**) FORS, (**C**) Raman SSE and (**D**) emission spectra (λ_exc_ = 435 nm) of three different areas (a), (b) and (c) as shown in the Vis detail (**B**). Additionally, it is reported the reference emission spectra of safflower red absorbed on paper.

Raman analyses evidenced first of all the use of red ochre, as shown, for example, by the spectrum in Figure 6 for a detail of Print 1, where the characteristic bands at 225, 295, 412 and 615 cm^−1^ [30,31] were observed. However, both Raman and fluorescence spectra obtained on different red details of the six prints, led to the recognition of other coloring materials besides red ochre. In particular, vermilion could be easily detected, thanks to its strong Raman signals at 252 and 342 cm^−1^ [31] (Figure 6). At the same time, a remarkable fluorescence signal was recorded with a maximum around 600 nm for various red areas upon excitation at 435 nm. It can be assigned to the red dye obtained from *Carthamus tinctorius*, i.e., safflower, based on the comparison of the emission spectra obtained for the details of the prints with those recorded for a reference sample of the dye absorbed on paper (Figure 6). The emission maximum for the red organic dye detected in the prints ranged from 596 to 605 cm^−1^, coherently with the wavelengths reported in the literature for safflower red spread in different binders on various supports [32]. The variation in the wavelength of the emission maximum and the fact that it was slightly higher than that of the reference sample (592 nm) are explained by the different concentrations of the dye, which can give rise to a more or less pronounced self-absorption effect [33].

It is interesting to note that the different spectroscopic techniques used for the analyses yielded, in more than one case, complementary results. For example, in the bright red detail of print 1 shown in Figure 6, the presence of vermilion was detected by Raman spectroscopy, while the bands in the FORS spectrum were mainly due to red ochre, even if its shape could suggest the contribution of safflower with the relative maximum at 460 nm and the flattening of the typical shape of the red ochre. In the same area, the emission spectrum confirmed the use of safflower red. While vermilion and red ochre obviously do not give rise to fluorescence emission, the red iron pigment must also be observed in the Raman spectrum, where, in contrast, just the bands of mercury sulfide were detected. For this reason, it should be assumed that the different results of the Raman and FORS techniques regarding the inorganic red pigments in this area, having a complex decorative pattern, were due to the different sizes of the measurement spots for the two techniques (see Section 2). Although less likely, their different penetration depth could also be considered, as the excitation wavelength used for Raman analyses, 830 nm, should penetrate deeper than the visible radiation in the FORS measurements and thus, in principle, the two pigments could also belong to different layers. In other cases, however, as in the red background of print 2, a true mixture of vermilion and red and perhaps also yellow ochre was demonstrated by the Raman spectrum, where the most intense bands due to HgS were accompanied by weaker signals at 290, 380 and 415 cm^−1^ suggesting the use of earth pigments [34] (Figure 6).

#### 3.3.2. Yellow Colors

**Figure 7:**
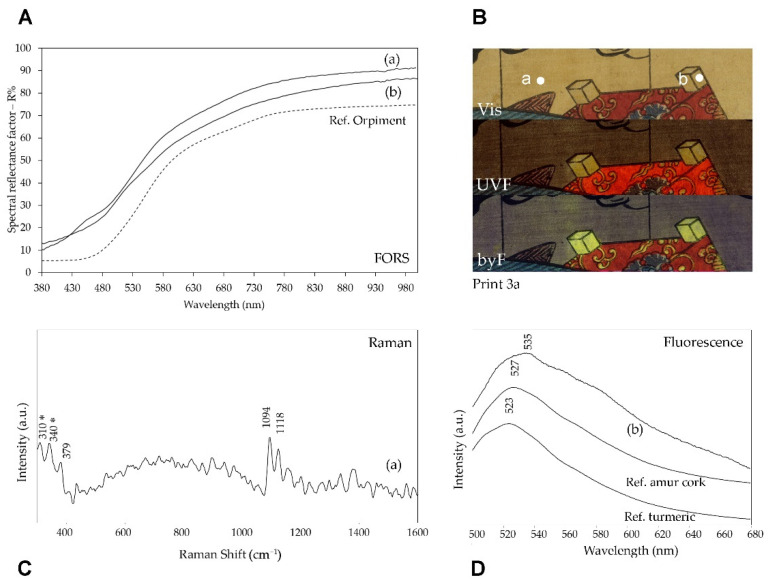
Yellow details in print 3a: (**A**) FORS, (**C**) Raman SSE and (**D**) emission spectra (λ_exc_ = 435 nm) of two different areas (a) and (b). These areas are shown in the upper right (**B**) as Vis and multiband fluorescence images. In the Raman SSE spectrum, bands marked with * are related to orpiment, while the remaining ones are due to paper (see Figure 8). In the lower right box, emission spectra are reported for area (b) and for reference samples of Amur cork dye and turmeric dye on silk.

The use of orpiment As_2_S_3_ was demonstrated in areas with different yellow shades thanks to the observation of the Raman bands at 310 and 340 cm^−1^, shown as an example in Figure 7 for the pinkish yellow background of print 3a. It is interesting to note that the band at 310 cm^−1^ corresponded well with the Raman spectrum reported in reference [34] for natural orpiment. The 340 cm^−1^ band was instead located at a lower wavenumber than the As_2_S_3_ mineral, suggesting a better correspondence with synthetic arsenic sulfide [35]. Similar features were observed in the Raman spectra of yellow or orange pigment particles in Hokusai prints dating back to the 1830s and were assigned to a synthetic arsenic sulfide prepared from natural orpiment as the main source of arsenic [36]. Apparently, a similar material was also found in these Kunisada prints, albeit dating to a later period, when a completely amorphous arsenic sulfide pigment was expected [36]. However, as discussed below, a different situation arose for the yellow pigment identified in the green colored areas.

Similarly to the red coloring materials, in addition to inorganic pigments, the use of an organic dye was also detected for yellow by means of spectrofluorimetry. For example, in print 3a, the yellow background where orpiment was identified by Raman spectroscopy did not yield a significant emission upon visible excitation at 435 nm, differently from the yellow details of the slats of the panier that the actor is carrying on his back. In fact, in agreement with the UVF and gbF images, the emission spectrum of the latter details shows a rather intense band with a maximum at about 535 nm (Figure 7). As shown in Figure S4, the multiband fluorescence image suggested the possible use of turmeric or Amur cork dye for these yellow areas, even if they could not be differentiated since their fluorescence emission had the same color for all the bands considered. The emission spectra obtained for reference samples of the two dyes (Figure 7) indicated a better correspondence with Amur cork, even if the maximum was at a lower wavelength for the reference in comparison with the print. This result was completely in agreement with the yellow-greenish fluorescence emission of the byF images in Figure 7. Unfortunately, the FORS spectrum did not help in the material differentiation since orpiment is the prevalent contribution to reflectance. This limitation is attributable to the peculiar printing technique with thin and overlapping layers of color, which can all contribute to reflectance.

#### 3.3.3. Blue Colors

**Figure 8:**
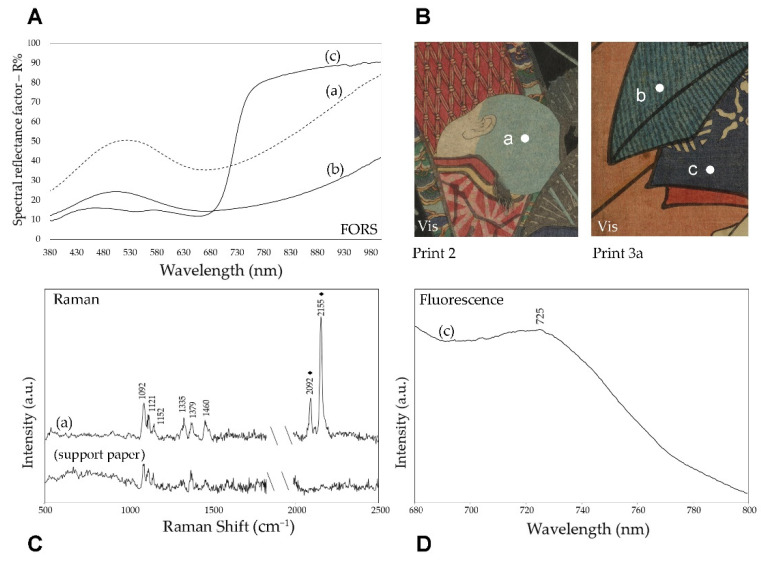
Blue details in print 2 and 3a: (**A**) FORS, (**C**) Raman SSE and (**D**) emission spectra (λ_exc_ = 562 nm) of three different areas (a), (b) and (c), as shown in the Vis detail (**B**). (♦) in the Raman SSE spectrum indicates the bands due to Prussian blue while the others are assigned to the support paper.

Raman spectroscopy also confirmed that Prussian blue was used in most areas with this color or a lighter shade, as indicated by the bands at 2092 and 2155 cm^−1^ (Figure 8). It is interesting to note that Prussian blue is the only pigment for which reflectance FTIR spectra also give unequivocal evidence, with a sharp band at 2096 cm^−1^ [37] (Figure S3). In parallel with the previously examined colors, spectrofluorimetry also demonstrated the use of the indigo organic dye for blue, with a characteristic emission maximum at around 725 nm [38] observed with visible excitation, as shown in Figure 8, for a detail of print 3a. Indigo was also used in a mixture with the same Prussian blue, as already documented for Japanese prints of the considered period [3]. It is, for example, the case of the dark blue background of print 4, where the signal of Prussian blue at 2096 cm^−1^ was observed in the FTIR spectrum (Figure S3), although with weak intensity, while the FORS spectrum clearly indicated the presence of indigo (Figure S5). As already reported in the literature, the combined use of different spectroscopic techniques is useful, or even necessary, when dealing with works containing mixtures of these two blue colorants [39].

**Figure 9:**
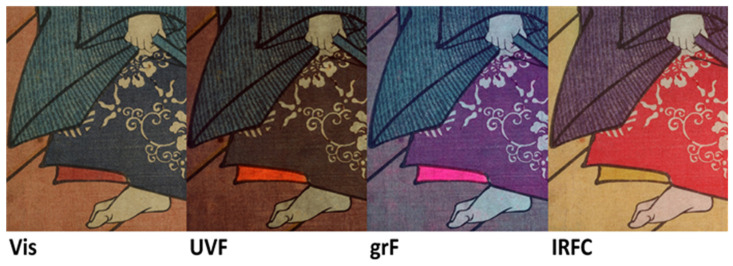
Print 3a, detail in Vis, UVF, grF and IRFC. Blue pigments in the theater costume of the actor show different responses as the band considered changes, allowing a characterization through comparison with spectroscopic data.

Multiband imaging can help the characterization and visualization of different blue pigments, allowing, in this case, the mapping of indigo and Prussian blues as reported in Figure 9.

#### 3.3.4. Green Colors

As already reported in the literature for prints by Kunisada himself and by Hokusai [3,36], in the present case, the use of mixtures of arsenic sulfide and Prussian blue for the green colors was also found. The blue pigment was easily identified, based on both the reflectance FTIR spectrum, as shown in Figure S3 for the dark green details of print 4, and the Raman spectrum, as in the case of the green background in the lower part of print 2 (Figure S6). The bands due to arsenic sulfide were also observed in the Raman spectrum, in addition to those at 275, 537, 2090 and 2153 cm^−1^ assigned to Prussian blue. Different from the above considerations of the yellow colors, in this case, the broad band centered at 330 cm^−1^ and those due to sulfur at 217 and 471 cm^−1^, suggested the use of an entirely amorphous, and thus synthetic, arsenic sulfide [35,36]. The hypothesis of the mixture was completely in agreement with the microscopic images of the analyzed areas, showing the presence of distinct yellow and blue particles. (Figure S7).

#### 3.3.5. Violet and Purple Colors

In the dark violet stripes of the actor’s coat in print 4, Prussian blue could be identified due to its characteristic bands around 2090 cm^−1^ in the FTIR spectrum and 2156 cm^−1^ in the Raman spectrum.

**Figure 10:**
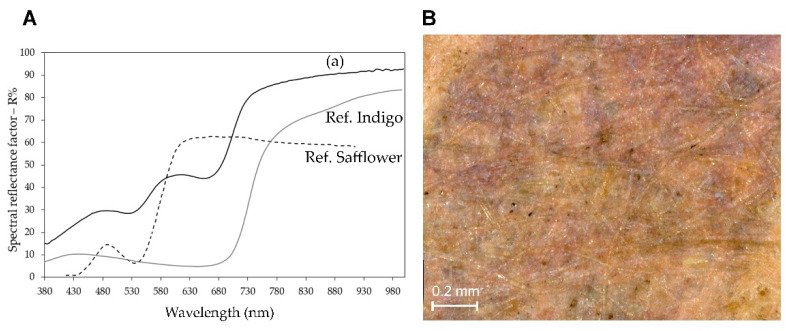
(**A**) FORS spectrum and (**B**) microscope image of a purple area in print 5. By analyzing FORS spectrum (a), we can assume the presence of a mixture made of blue and red pigments such as indigo and safflower. In the microscope image, we can also see blue and red areas supporting this hypothesis.

In the purple details of print 5, however, the presence of safflower red was positively determined based on the emission spectrum excited at 435 nm, which showed a maximum at 580 nm (Figure S8). It should be noted that this wavelength was slightly lower than those reported above for the same dye in the red areas of the prints, but also in this case the observed variation could be explained through a self-absorption phenomenon due to the blue component, which is necessary to obtain the purple color, and obviously gave rise to an absorption in the red region of the spectrum. The use of safflower red in a mixture with a blue dye or pigment is widely reported in the literature for purple in Japanese prints, in particular from the 1830s to 1864, after which synthetic purple dyes were introduced [2]. Dayflower was in most cases the blue substance with which safflower red was mixed, and this combination of dyes has been detected for Kunisada prints dating back to the 1850s [3,4]. In other cases, indigo was used and more rarely Prussian blue [2,15]. By analyzing the maxima in the FORS spectrum (Figure 10), we could exclude the presence of dayflower and confirm that of indigo as a blue component in the mixture [40].

#### 3.3.6. Black, Grey and Brown Colors

The characteristic broad Raman bands around 1350 and 1580 cm^−1^ indicated the presence of amorphous carbon as black pigments in the brown areas of prints 3b and 5 and the greyish details of print 5 [34] (Figure S9). Near infrared images confirmed the presence of carbon-based black pigments in all the black areas (thin lines, contours, writings and background) while the brown area in print 3b appeared to have been made of a mixture of red ochre and carbon black.

## 4. Conclusions

This non-invasive in situ multidisciplinary campaign yielded important information in the context of the specific conservation and musealization project for these six woodblock prints, that had never been studied before. The combination of all the techniques applied in this study represents a set of complete information useful to the different professionals involved: historians, conservators and curators in the museum.

The results of the analyses enabled a more targeted approach to the restoration intervention and the preservation of the prints over time; the treatments were calibrated through knowledge of their surface morphology, while identification was made of pigments and colorants such as Prussian blue and safflower red that are particularly sensitive to a basic pH environment. Thanks to their characterization, deacidification treatments and the use of housing materials with alkaline reserve could be avoided. In addition, also the lightfastness of the materials present in the woodblock prints was evaluated for the best conservation.

The materials identified were completely in agreement with those traditionally used in the Edo period in the 19th century and the results add new data to expand the knowledge of techniques and materials in Japanese woodblock printing.

A new approach was proposed by adding multiband imaging techniques to single-point spectroscopic data, thus obtaining detailed information on the distribution of the different materials in each print.

The computational imaging technique of RTI, apart from giving useful data on the paper conservation conditions, gave an additional amount of information in terms of characterization of the printing techniques, which could not be obtained otherwise.

Since the objects studied belong to the civic Museum, and so to the whole community, the outcomes of this research will be useful both in scientific studies of the artworks, and as additional resources in temporary exhibitions and cultural events for a wide public, supporting the Museum’s mission to disseminate knowledge on Japanese art and Japanese culture.

## Figures and Tables

**Table 1:** The woodblock prints by Utagawa Kunisada investigated by imaging and spectroscopic techniques.

Print		Date	Brief Description
**1**	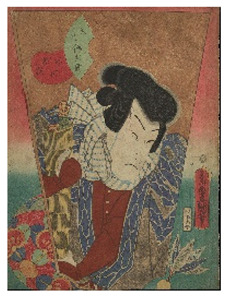	1862	Actor Ichimura Kakitsu IV as Tenjiku Tokubei (*hagoita* battledore picture).
**2**	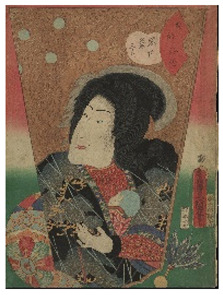	1862	Actor Iwai Kumesaburō III as Kijin no Omatsu (*hagoita* battledore picture).
**3 a**	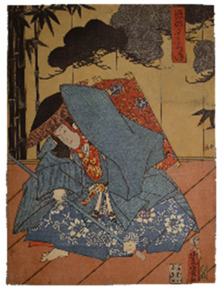	1859	Triptych (missing character on the right). Representation of the play *Kanjinchō*; actor Iwai Kumesaburō III as Minamoto no Yoshitsune (left); actor Kawarazaki Gonjūrō I as Musashibō Benkei (center).
**3 b**	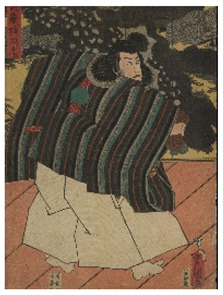	1859	
**4**	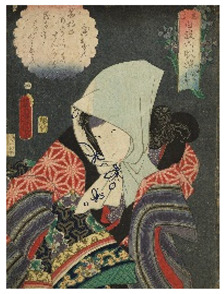	1861	The Imitation Komachi (Gisu Komachi); actor Sawamura Tanosuke II as Mishima Oesen. From the series Selected Underworld Characters for the Six Poetic Immortals (*Mitate shiranami rokkasen*).
**5**	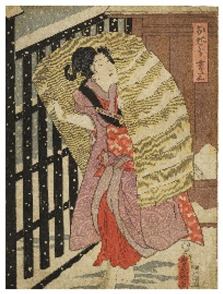	1859	Triptych/diptych? Representation of the play *Sannin Kichisa Kuruwa no Hatsugai*; actor Iwai Kumesaburō III as Ojō Kichisa.

**Table 2:** The methods of imaging acquisition with specifications of excitation (λ_exc_), acquisition (λ_acq_) wavelengths and abbreviations used in the paper.

Methods of Acquisition	Abbreviation	λ_exc_ (nm)	λ_acq_ (nm)
Visible reflected	Vis	380–780	380–780
UV reflected	UVR	365	350–395
UV false color	UVFC	365–600	365–600
Visible fluorescence induced by UV emission	UVF	365	>420
Visible fluorescence induced by visible radiation	byF	440	>480
	bgF	440	>520
	grF	530	>600
Near infrared reflected	NIR	>850	850–1000
Infrared false color	IRFC	420–1000	420–1000

**Table 3:** Coloring materials identified in the six woodblock prints by Utagawa Kunisada examined in the present work.

Color	Identified Material	Print					
		1	2	3a	3b	4	5
**Red**	Vermillion	x	x			x	
	Safflower	x			x		x
	Red ochre	x	x	x	x		
**Yellow**	Yellow ochre	x		x	x	x	x
	Orpiment	x	x	x	x		
	Organic dye	x		x		x	
**Blue**	Prussian blue	x	x	x		x	
	Indigo			x			
**Green**	Prussian blue + orpiment	x	x		x	x	
**Purple**	Indigo + safflower					x	
**Brown**	Red ochre + carbon black				x		
**Black**	Lamp black	x	x	x	x	x	x

## Data Availability

Not applicable.

## Supplementary Materials

The following supporting information can be downloaded at: https://www.mdpi.com/article/10.3390/s22228772/s1, Figure S1: Comparison of Vis (left) and RTI elaboration (right) of a detail in print 3b; Figure S2: Comparison of Vis (left) and RTI elaboration (right) of a detail in print 4; Figure S3: FTIR spectra acquired in external reflection mode for: (a) a green detail in print 4; (b) a light blue detail in print 3b; (c) an uncolored area in print 2; Figure S4: Multiband fluorescence image of five yellow dyes used in Japanese woodblock printing; Figure S5: FORS spectra acquired in correspondence to the dark blue background of print 4; Figure S6: Raman SSE spectrum acquired on the green background of print 2; Figure S7: Optical microscope image (250×) of a green area where blue and yellow separate areas are visible; Figure S8: Emission spectrum (λexc = 435 nm) of a purple detail in print 5; Figure S9: Raman SSE spectra of a brown detail of print 3b and brown and grey details of print 5.
