# Supplementary material for: From Materials to Technique: A Complete Non-Invasive Investigation of a Group of Six Ukiyo-E Japanese Woodblock Prints of the Oriental Art Museum E. Chiossone (Genoa, Italy)
Source: Sensors (Basel). 2022 Nov 13;22(22):8772. doi: 10.3390/s22228772 (PMC9693042; doi:10.3390/s22228772)
Supplement: Supplementary file 1 [file sensors-22-08772-s001.zip › sensors-1991285-supplementary.pdf]

Supplementary Materials of "From materials to technique: a complete non-invasive investi-gation of a group of six ukiyo-e Japanese woodblock prints of the Oriental Art Museum E. Chiossone (Genoa, Italy)" by M. Gargano, M. Longoni, V. Pesce, M. C. Palandri, A. Canepari, N. Ludwig, and S. Bruni

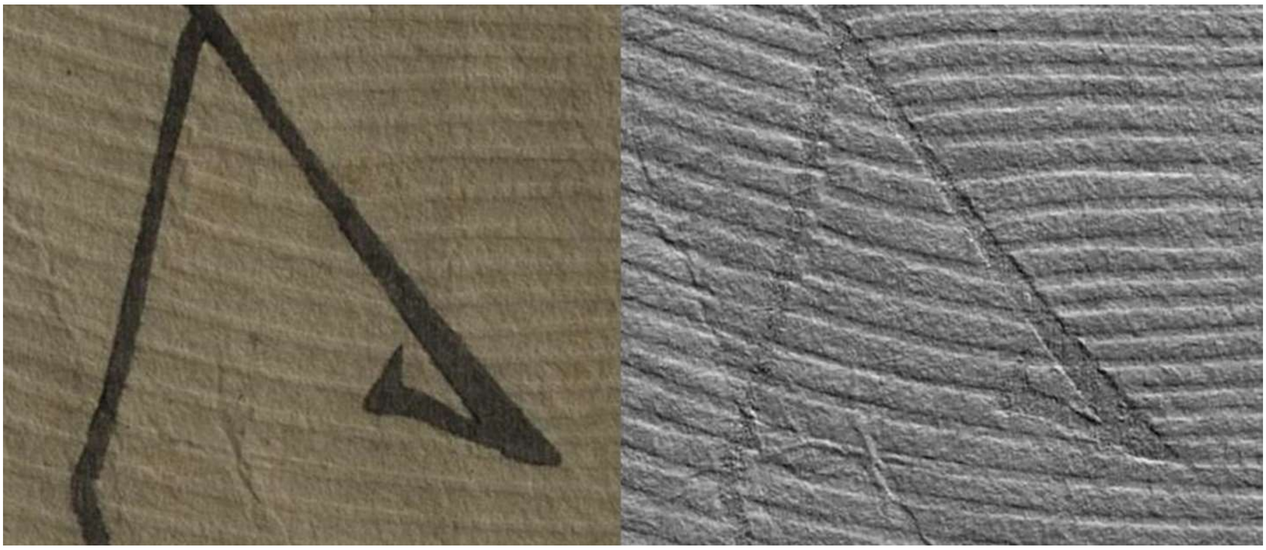

Figure S1. Comparison of Vis (left) and RTI elaboration (right) of a detail in print 3b. RTI technique in this case is able to highlights the *Karazuri* printing technique, literally "empty printing". This technique is realized carving a pattern into a woodblock and then printing it in the usual way with a *baren*, but without any pigment being applied to the wood.

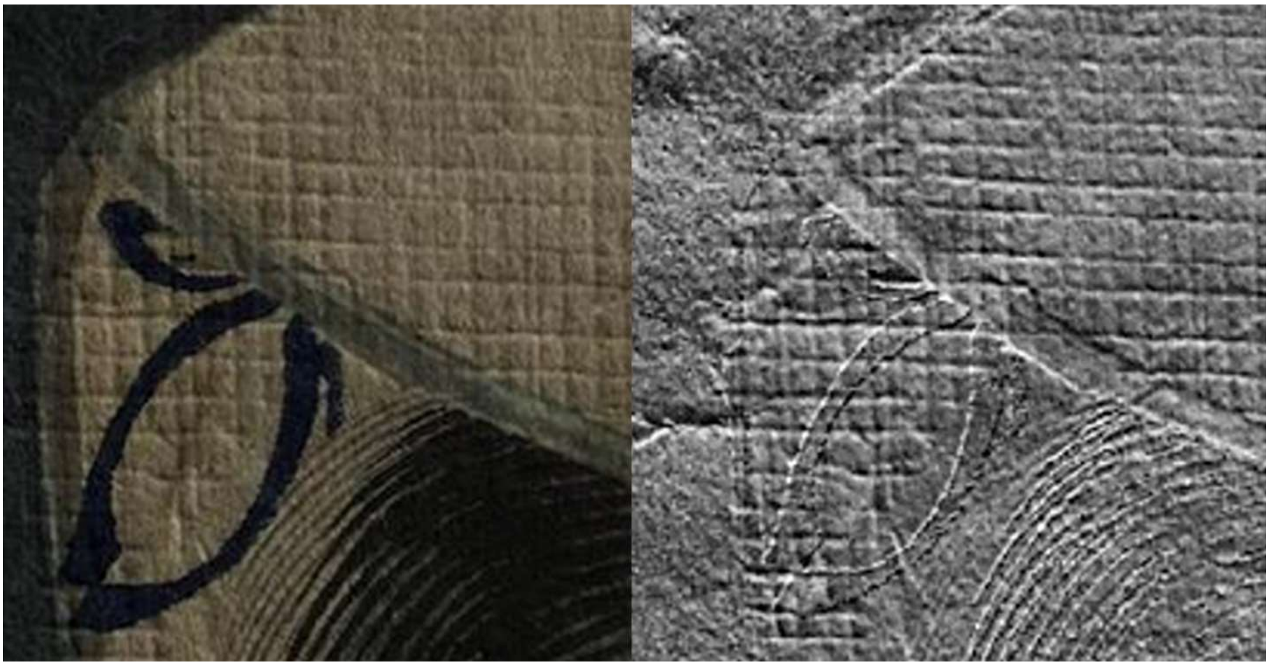

Figure S2. Comparison of Vis (left) and RTI elaboration (right) of a detail in print 4. RTI technique in this case is able to highlights the *Nunomezuri* technique where a piece of fabric is glued to a woodblock and through the pressure applied by the *baren* the texture of the fabric is embossed on the paper.

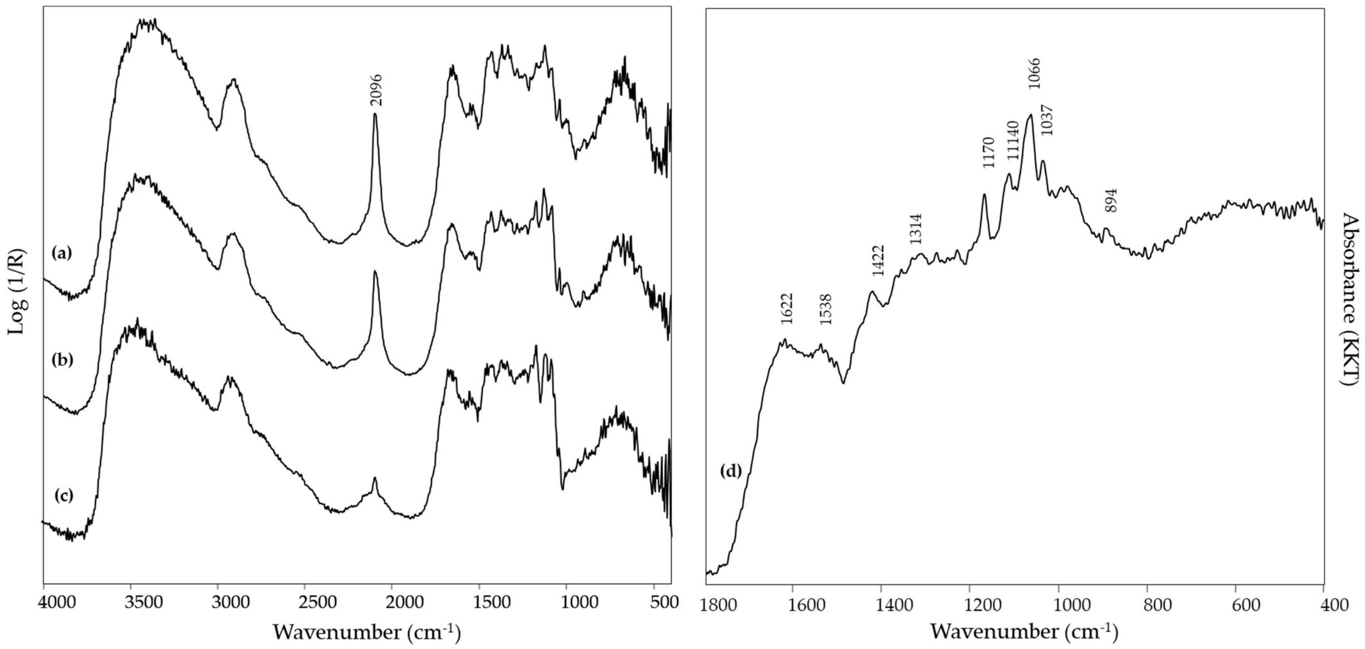

Figure S3. FTIR spectra acquired in external reflection mode for: (a) a green detail in print 4; (b) a light blue detail in print 3b; (c) the dark blue background of print 4; (d) an uncolored area in print 2. Spectra (a), (b) and (c) are shown as pseudo-absorbance log(1/R), while spectrum (d) has been obtained from the Kramers-Kronig transform applied to the reflectance data. In (a), (b) and (c) the marked peak at 2096 cm^{-1} is assigned to Prussian blue, in (d) the marked peaks are attributed to cellulose.

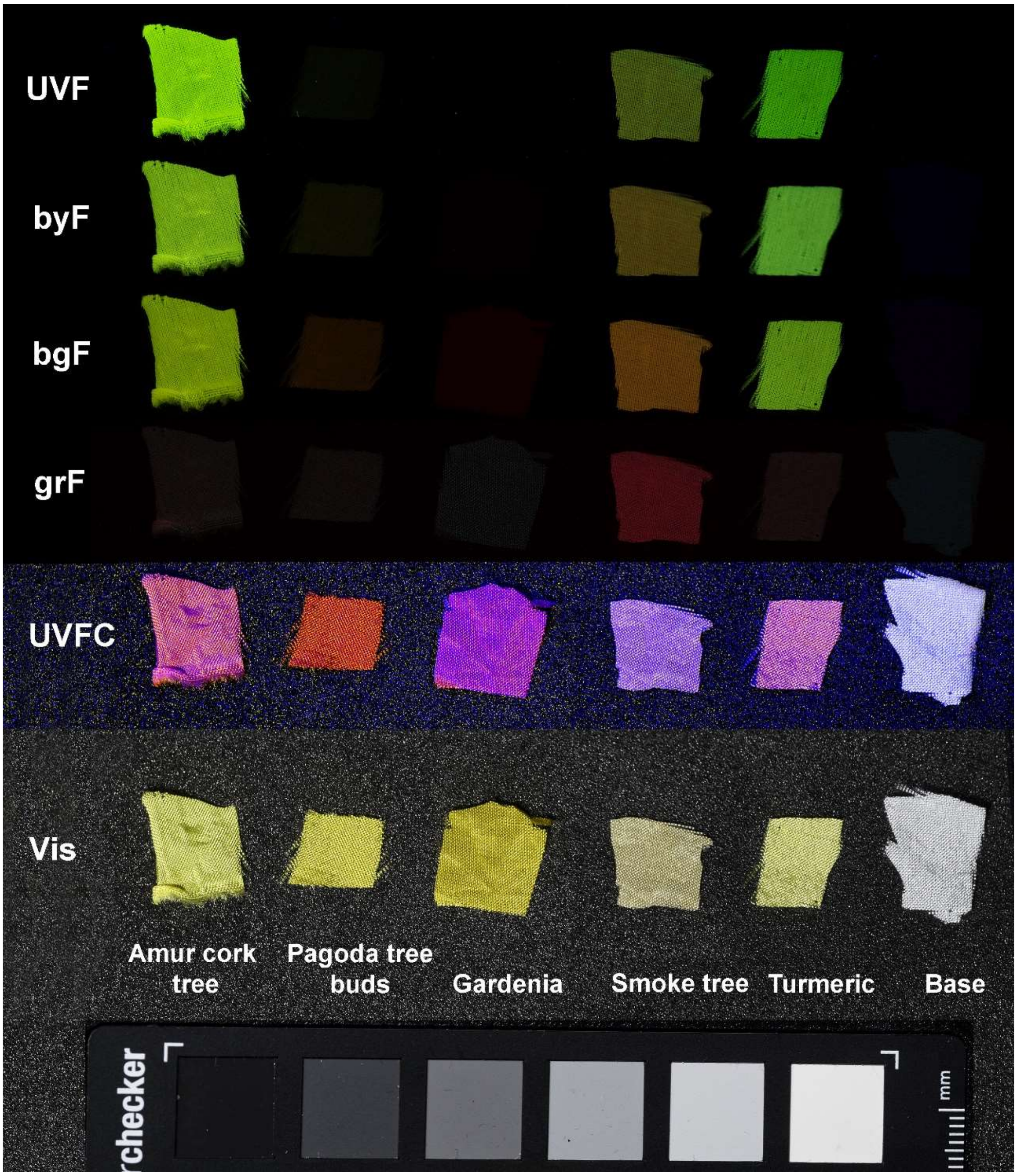

Figure S4. Multiband fluorescence image of five yellow dyes used in Japanese woodblock printing. The result of this technique allows to exclude the presence of Pagoda tree, Gardenia and Smoke tree dyes from the use in the considered woodblock prints. Unfortunately, since the fluorescence of Amur cork tree and turmeric is very similar, it is not possible to differentiate the two materials.

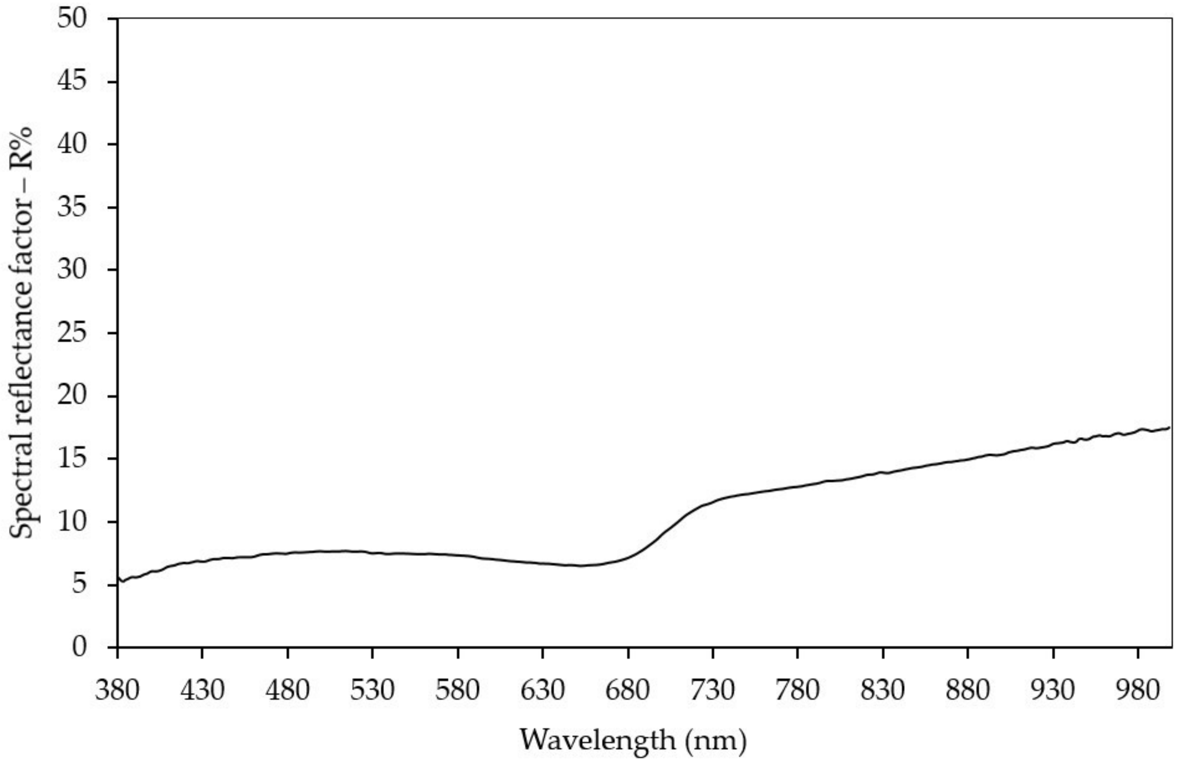

Figure S5. FORS spectra acquired in correspondence of the dark blue background of print 4 where it is possible to find the presence of indigo together with Prussian blue found using FTIR (Figure S3).

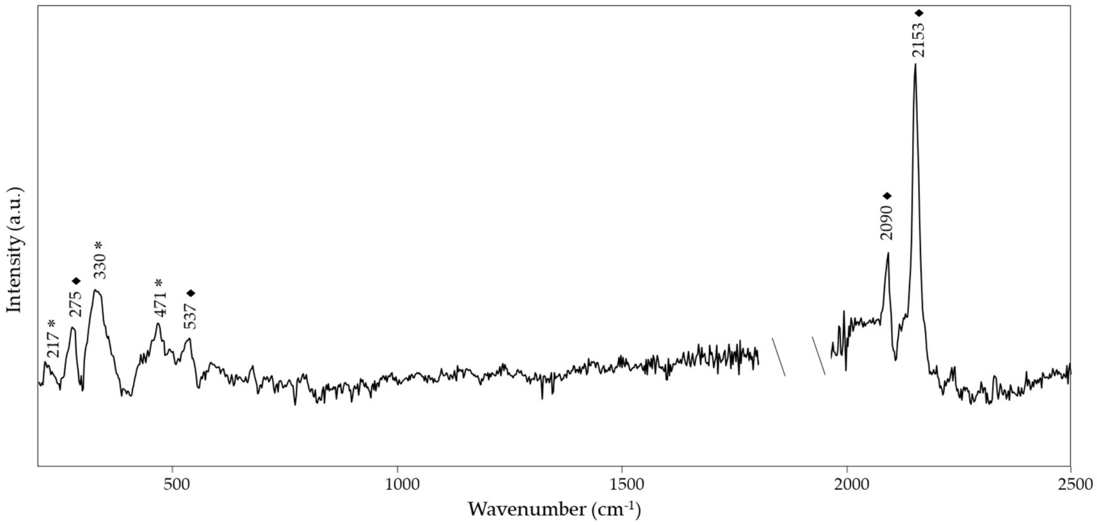

Figure S6. Raman SSE™ spectrum acquired on the green background of print 2. Legend: ∗ = band due arsenic sulfide, ◆ = band due to Prussian blue.

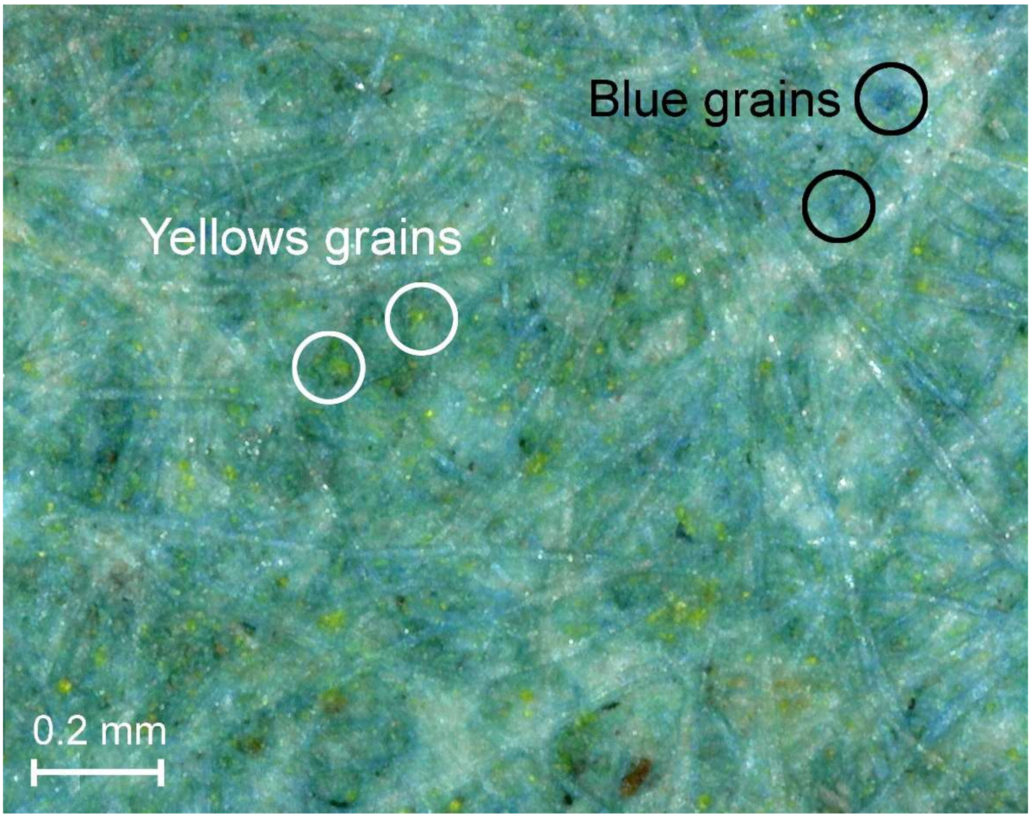

Figure S7. Optical microscope image (250x) of a green area where blue and yellow separate areas are visible. Spectroscopic technique allowed to identify the presence of Prussian blue and orpiment.

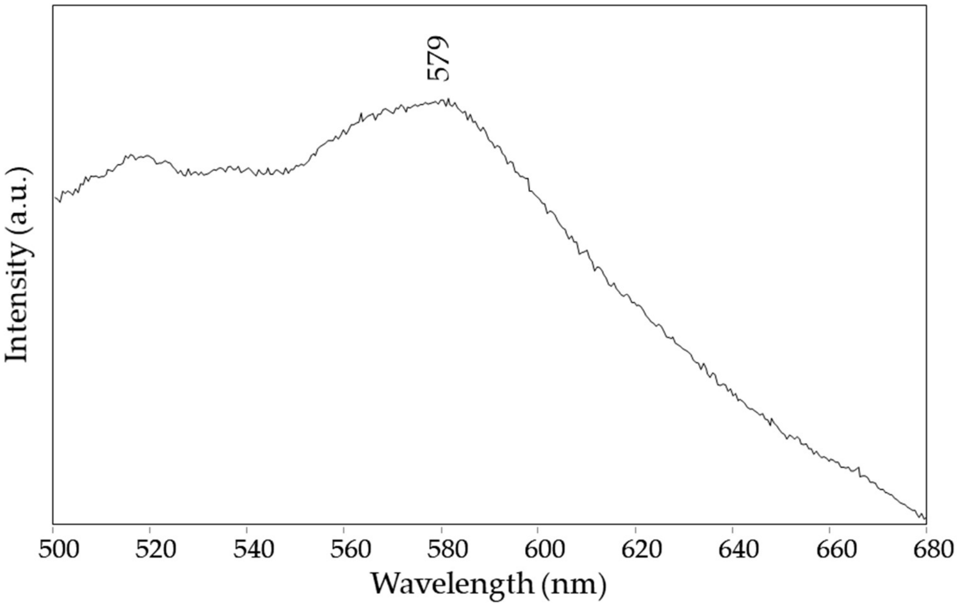

Figure S8. Emission spectrum (λ_{exc} = 435 nm) acquired on the purple kimono of the actor in print 5.

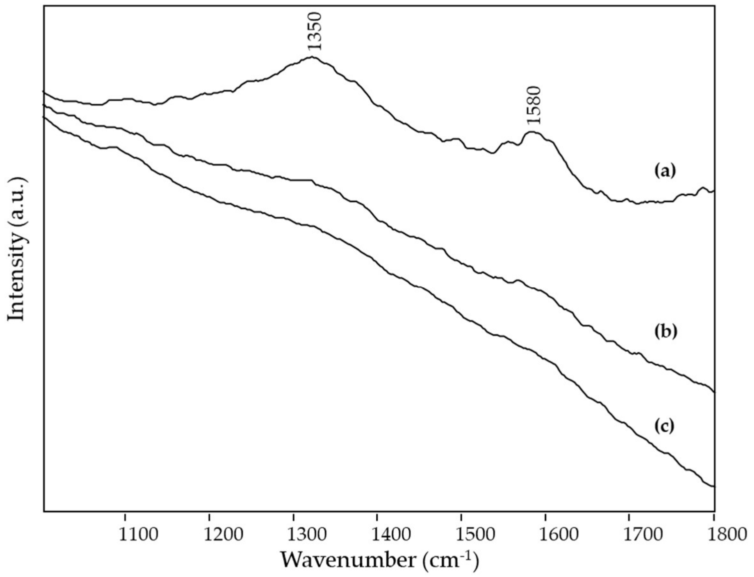

Figure S9. Raman SSE™ spectra of: (a) brown detail of print 3b; (b) brown detail of print 5; (c) grey detail of print 5.
